# Numerical Investigation of the Orientability of Single Reinforcement Fibers in Polymer Matrices

**DOI:** 10.3390/polym14030534

**Published:** 2022-01-28

**Authors:** Anja Winkler, Niels Modler, Maik Gude, Yun Xu, Martin Helwig, Eike Dohmen, Axel Dittes, Dominik Höhlich, Thomas Lampke

**Affiliations:** 1 Institute of Lightweight Engineering and Polymer Technology (ILK), Technische Universität Dresden, 01307 Dresden, Germany; niels.modler@tu-dresden.de (N.M.); maik.gude@tu-dresden.de (M.G.); Yun.Xu@tu-dresden.de (Y.X.); martin.helwig@tu-dresden.de (M.H.); 2 Institute of Mechatronic Engineering (IME), Technische Universität Dresden, 01069 Dresden, Germany; eike.dohmen@tu-dresden.de; 3 Institute of Materials Science and Engineering (IWW), Chemnitz University of Technology, 09107 Chemnitz, Germany; axel.dittes@mb.tu-chemnitz.de (A.D.); dominik.hoehlich@mb.tu-chemnitz.de (D.H.); thomas.lampke@mb.tu-chemnitz.de (T.L.)

**Keywords:** fiber-reinforced polymers, magnetic fiber alignment, polymer matrix, magnetic permeability, fiber length, magnetic coating, magnetic field, simulation

## Abstract

Fiber-reinforced polymers are increasingly being used, especially in lightweight structures. Here, the effective adaptation of mechanical or physical properties to the necessary application or manufacturing requirements plays an important role. In this context, the alignment of reinforcing fibers is often hindered by manufacturing aspects. To achieve graded or locally adjusted alignment of different fiber lengths, common manufacturing technologies such as injection molding or compression molding need to be supported by the external non-mechanical process. Magnetic or electrostatic fields seem to be particularly suitable for this purpose. The present work shows a first simulation study of the alignment of magnetic particles in polymer matrices as a function of different parameters. The parameters studied are the viscosity of the surrounding polymer as a function of the focused processing methods, the fiber length, the thickness and permeability of the magnetic fiber coatings, and the magnetic flux density. The novelty of the presented works is in the development of an advanced simulation model that allows the simulative representation and reveal of the fluid–structure interaction, the influences of these parameters on the inducible magnetic torque and fiber alignment of a single fiber. Accordingly, the greatest influence on fiber alignment is caused by the magnetic flux density and the coating material.

## 1. Introduction

Short and long carbon fibers have excellent mechanical properties, which make them a suitable reinforcement for polymer composites. Injection molding and press molding are two common methods of processing short and long-fiber thermoplastic composites. In such processes, the fibers are usually unoriented. Appropriate alignment of fibers can improve their physical properties for injection molding and press molding processes [1,2].

For both injection molding and press molding processes, traditional methods for optimizing continuous fiber orientation, such as multiaxial fabrics or tailored fiber placement (TFP) [3], are no longer applicable to produce short and long-fiber composite components with a gradual fiber distribution. In this context, the possibility of aligning the reinforcing fibers using external action mechanisms is considered. Through external mechanisms of action, it is possible to produce composite components with the desired fiber distribution, such as different fiber lengths over the cross-section or fibers oriented in one direction in components.

There are different methods of external action mechanisms to produce composites with aligned short and long fibers, including the application of an electric [4], magnetic [5,6], the combination of magnetic [7], or ultrasonic field [8]. The most common method of aligning fibers among them is by electric or magnetic fields. However, the magnetic field has several advantages that distinguish it from the electric field or other techniques. Firstly, magnetic fields are non-contact volumetric forces that do not cause any chemical change in the material. Secondly, permanent magnets and electromagnets are readily available today and produce sufficiently strong fields to align the fibers and finally, magnetic fields are not as dangerous as electric fields. For this reason, the initial investigations for the fiber alignment presented in this study focus on the use of the magnetic field.

As can be seen from the types of possible reinforcing fibers [9,10,11,12,13,14,15,16,17], not only the mechanical properties but also the electrical conductivity and magnetic properties of a fiber can be improved by a coating with Nickel, Iron, or Cobalt [18,19]. Thus, coated carbon fibers can be even more easily aligned in an electric or magnetic field. For this reason, coated fibers are investigated in the present work.

## 2. State of the Art—Magnetic Alignment of Reinforcement Fibers

Ciambella [5] investigates the alignment of Ni-Coated (NiC) carbon fibers (diameter 4.8 µm, fiber length 0.25 mm) in a polydimethylsiloxane (PDMS) matrix using a magnetic field. Specifically, the work investigates the influence of the viscosity of the matrix, the magnetic flux density, and the aspect ratio (length/diameter ratio) on the alignment. Stanier [6] successfully fabricated short PDMS composites reinforced by NiC carbon fibers. The NiC carbon fibers were aligned in a matrix with a viscosity of 5.5 Pa·s using a weak magnetic field. Timbrell [9] dealt with the alignment of carbon fibers and other Iron-coated man-made fibers in liquid suspension using a magnetic field. The alignment of fibers depends on the fiber diameter, the fiber length as well as on the density, and viscosity of the matrix, and the strength of the magnetic field. A study presented by Chung [10] investigated magnet-assisted injection molding for the orientation of NiC fibers during the melt polymer filling process. Hatta [16] theoretically derives which preconditions have to be fulfilled to selectively align a Nickel-coated graphite fiber in a fixed time. Based on the derived preconditions, several parametric studies were performed. In the paper of Pishvar [12], the orientation of NiC carbon fibers was investigated with the aid of a magnetic field during the curing of epoxy resin. The effects of different magnetic flux densities (10 mT to 50 mT), fiber lengths (0.1 mm and 0.25 mm), and Nickel coating content (20% by weight and 40% by weight of the fiber) on the alignment behavior of NiC fibers were discussed. At a magnetic field of 50 mT, in addition to a rotation and network formation of the fibers, a significant migration of the fibers with a length of 0.1 mm towards the magnetic pole can be observed. In addition, the longer fibers (0.25 mm) achieve better alignment at the same magnetic flux density (30 mT).

In summary, previous published works on fiber alignment mainly focused on experimental and theoretical studies on the alignment of fibers. In the case of experimental studies, the number and range of influencing parameters are often limited. For example, previous works only focus on very comparatively short fibers, but there are almost no studies on long fibers. Only a few attempts were made to analyze the fiber alignment by using a finite element method (FEM) simulation. Furthermore, FEM simulations for studying the effect of fiber and magnetic field parameters on fiber alignment are almost non-existent. However, parameters such as magnetic flux density, coating thickness [2], fiber length [20], coating material, and the viscosity of the polymeric matrix [5] have a huge influence on the alignment behavior of short and long-fibers. The work in this paper is intended to gain a deeper understanding of the occurring effects by taking the known relationships into account. For this purpose, the simulation parameters, as well as a test plan for the simulation, are selected. A suitable multi-physical simulation model is set up and described using the finite element method (FEM) COMSOL software (V5.4, COMSOL Inc., Stockholm, Sweden). Thus, parameter studies are carried out to simulate the fiber alignment process. Finally, the test results are evaluated and compared to each other and used for determining the effect of parameters on the fiber alignment process.

## 3. Materials and Methods

### 3.1. Material

**Figure 1:**
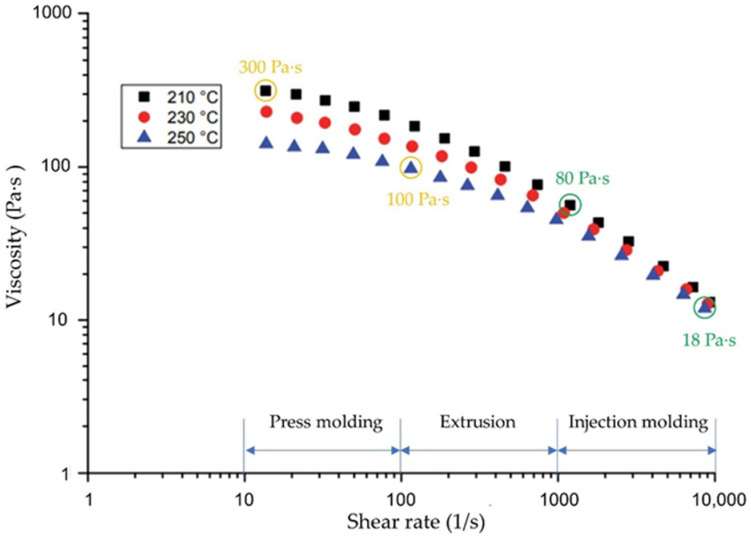
Temperature and shear rate dependent rheological behavior of PP in relation to typical manufacturing processes for fiber-reinforced composites (press molding, extrusion and injection molding) in accordance with [22].

Previous work has pointed out that magnetic flux density, coating thickness, coating material, fiber length, and the viscosity of the matrix have a huge influence on the alignment behavior of reinforcement fibers. For thermoplastic matrices, the rheological behavior is described by flow curves [21]. Figure 1 shows the flow behavior in form of the viscosity of polypropylene (PP) for different processing temperatures and shear rates.

It is shown that the viscosity of PP at the processing temperatures commonly used for injection molding is in the range of 18 Pa·s to 80 Pa·s. For press molding processes it is in the range of 100 Pa·s to 300 Pa·s. Additionally, the viscosity of a polymer depends on the respective temperature and the exposed shear rate. In the laboratory, it proves difficult to control the stability of these two values. Even a slight change in polymer viscosity sometimes results in high deviations in the test results. Similarly, it proves to be very challenging to align the fiber in the molten polymer at more than 200 °C, which will therefore only be investigated in later tests and not in the present work. For the investigations carried out, the viscosity of the molten polymer is replaced by the equivalent viscosity of different silicone oils at room temperature. In this way, both a defined viscosity that is almost constant throughout the experiment and the feasibility of the experiment can be ensured. A total of nine different silicone oils were selected, which are suitable to compare the viscosity of PP during press molding and injection molding. Due to the fact that the dwell time of the magnetic field is one of the main influences for the rearrangement of the fibers, the influence of pulsed magnetic excitation will be investigated in further experimental studies. The selected oils, designated as S1 to S9, and the respective kinematic and dynamic viscosities are listed in Table 1. The silicon oils are obtained from Quax GmbH (Otzberg, Germany).

**Figure 2:**
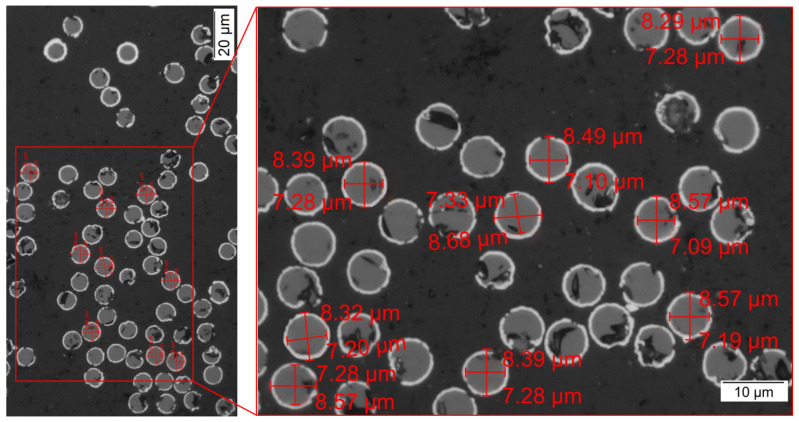
Cross-sectional analysis for the determination of fiber diameter and coating thickness—micrograph of sample 01-01.

According to Zhang [24], the shape of a fiber influences its properties. Likewise, the fiber shape is also important for the orientation of the fibers. Before the simulation experiment, the shape of the fibers has been determined by microscopic analysis. Thereby, Nickel-coated fibers of Toho Tenax HTS 40 MC 12 K 1420 Tex with a circular cross-section were investigated. These fibers are obtained from inca-fiber GmbH (Chemnitz, Germany) by galvanic Nickel metallization of standard carbon fiber rovings. Thus, a cylindrical shape of short and long fibers is taken as a basis for the simulation within the scope of the present work. The fiber diameter and coating thickness are then determined by micrograph analysis of the fiber cross-section. Figure 2 shows the microscopic measurements of an exemplary sample (sample 01-01). Thus, a determined mean value of the carbon fiber diameter of 7.2 μm is taken for the definition of the fiber diameter in the simulation.

For the orientation of fibers using magnetic fields, the relative permeability of the fiber material or the fiber coating material is also a very important parameter. For the performed studies, coated fibers are investigated. Thus, the force on the fiber in the magnetic field is related to the relative permeability of the coating. In the current work, the coating material is assumed to be a linear magnetic material. The magnetic properties are only approximated by a constant permeability. Table 2 shows the maximum magnetic relative permeability for common coating materials. Exemplary materials, which represent the permeability ranges from 100–1000, 1000–10,000 and above 10,000 were selected. Thus, three common materials—Nickel (500), Iron (5000) and Cobalt–Iron (18,000)—are selected as coating materials for the parameterized simulation studies. Hajjari [25] indicates that a coating thickness of 0.5 μm allows the most uniform coating of the entire fiber surface and the best continuity on carbon fibers. When the coating thickness falls below 0.2 μm, the discontinuity of the coating becomes prominent. If the coating thickness exceeds 1 μm, the toughness of the fibers decreases significantly. For this reason, a coating thickness of 0.2 µm to 1 μm was selected for parametric simulation studies.

Short fibers are assumed as fibers with a length of 100 µm to 1000 μm, while long fibers are characterized by a length of 1000 µm to 50,000 μm [31]. Regarding identifying the influence of fiber-length, a short fiber length of 100 μm to 1000 μm and range of 1000 μm to 10,000 μm was selected for the simulation studies. Additionally, the flux density of the magnetic field ranges from 20 mT to 1000 mT. All investigated parameters and the range of their values for the simulation are listed in Table 3.

### 3.2. Simulation Methods

**Figure 3:**
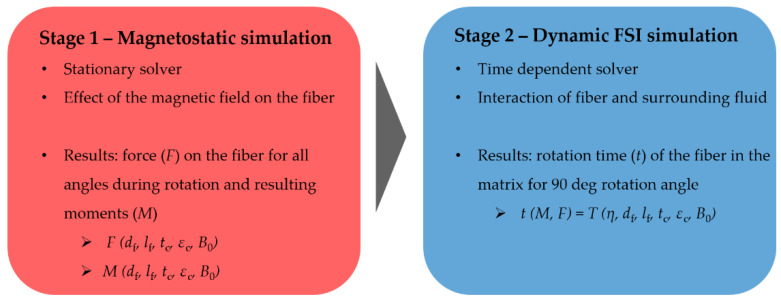
Developed simulation procedure divided into two stages for the representation of the induction of a force caused by an external magnetic field to the fiber (stage 1) and the interaction of the fiber and surrounding fluid during the orientation process (stage 2).

The orientation of fibers in a fluid using magnetic fields is a multiphysics problem, where the following two research areas are involved: electromagnetics and fluid–structure interaction (FSI). On the one hand, the loading of the fibers by magnetic fields, and on the other hand, the interaction of the fibers with a fluid have to be considered. In this work, the simulation and model setup is performed using COMSOL (V5.4, COMSOL Inc., Stockholm, Sweden). To establish a suitable physical model, the simulation is divided into two stages (see Figure 3). The first stage comprises the magnetostatic simulation, where the force on the fiber is calculated at each angle during rotation. The calculation results (forces) of the static magnet simulation act as input values for the second simulation stage. Here, the dynamic FSI simulation is performed, where the rotation of the fiber in the polymeric matrix is calculated. Figure 3 shows the developed procedure for the simulation of the coupled electromagnetic and fluid–structure interaction simulation.

**Figure 4:**
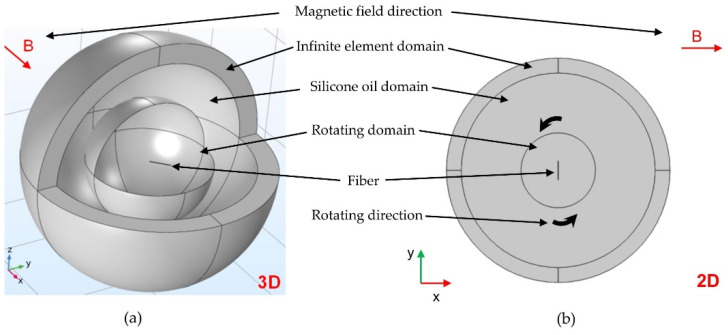
Reduction in the simulation model: (**a**) 3D model; (**b**) simplified 2D model.

Regarding the rotation of the fiber in the matrix using a magnetic field, the rotation of the fiber in the 3D space can be considered as a 2D rotation in the fiber rotation plane (Figure 4). The fiber’s physical thickness (i.e., z-direction) is parameterizable via the model depth, which scales all 2D volume-related simulation results. In order to approximate the 3D simulation with the simplified 2D model, an appropriate depth based on the diameter of the fiber can be derived.

As the complex 3D motion can be simplified to a 2D motion, a 2D model is used in the presented work. By using a 2D model instead of 3D solid elements, a representative model with fewer nodes can be created. Although some accuracy is sacrificed, the computation time of the simulation is significantly reduced.

The movement of multi-fibers in fluids is complex and will be investigated in future studies. This paper focuses on the movement of single fibers in the fluid. The time which is necessary to achieve a single fiber motion in the liquid is investigated. Since the fluid is not given an external velocity in the simulation, additional external loads, such as external pressure loading and shear rate during injection molding, are also not taken into consideration in these early investigations. The alignment of a single fiber will be observed in a constant time static magnetic field. This is because it is only the motion of the single fiber that needs to be concerned, not the change in the magnetic field. The magnetic field around the fiber can be described by magnetostatics with no free currents.

**Figure 5:**
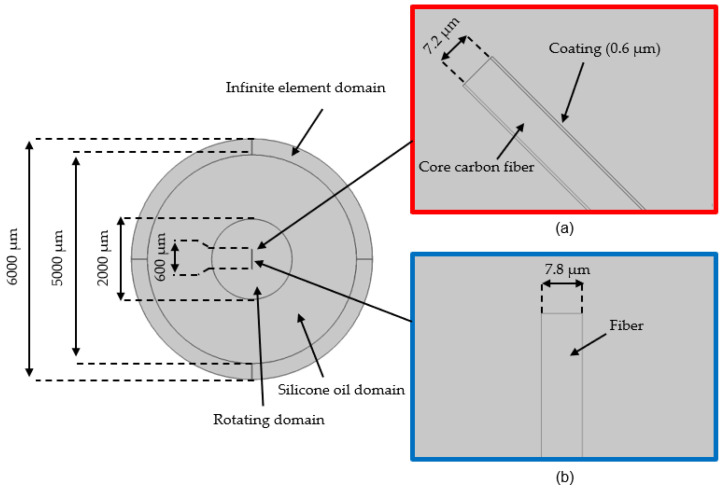
The geometry of the simulation model with coating thickness 0.6 µm and fiber length 600 µm: (**a**) stage 1—magnetostatic simulation; (**b**) stage 2—dynamic FSI simulation.

The developed geometric model consists of four components, including an infinite element domain, a silicone oil domain, a rotating domain, and a fiber (see Figure 5).

In the first simulation stage (magnetostatic), only the influence of the magnetic field on the fiber is considered. The silicone oil domain and the rotating domain are filled with air. The fiber is placed in the center. The force on the fiber in the magnetic field results mainly from the magnetic coating material. Therefore, the geometry of the fiber is divided into two parts, the core fiber, and the coating, as shown in Figure 5a. In the second stage, only the influence of the FSI is considered in the dynamic study. The silicone oil domain and the rotating domain are filled with fluid and the magnetostatic simulation does not need any consideration in this case. Thus, the fiber does not have a coating layer in the second stage, as shown in Figure 5b.

The calculation effort for the magnetostatic simulation is less than that for the transient FSI simulation. Additionally, even with a fine-meshed geometry, the computational effort of simulation stage 1 is not too high. Therefore, the model in stage 1, in particular for the narrow area of the coating, can be meshed with smaller element sizes to obtain a more precise input value for simulation stage 2. Thus, a mesh consisting of a total of 38,000 triangular elements was chosen for simulation stage 1. For simulation stage 2, a high computational effort is required if the meshing of the model is not optimized. Especially regarding the rotating domain and the fiber, the meshing has to be refined. After a convergence study of the mesh, a mesh consisting of 16,218 triangular elements was chosen for simulation stage 2.

For a situation where no free currents exist, but only a static magnetic field, simplified Maxwell–Ampère law of magnetostatics and Gauss’s magnetic law can be used to obtain the magnetic field around the fiber by the following equations:

(1) ∇×H=0

(2) ∇·B=0

**Figure 6:**
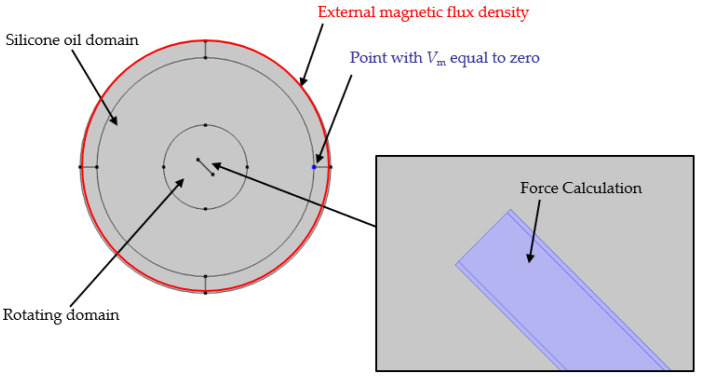
Settings of the boundary conditions and the load for the magnetostatic simulation (stage 1).

There, *B* is the magnetic flux density and *H* is the magnetic field intensity. The fact that the magnetic intensity field is irrotational (curl-free) means that a scalar potential as following exists:

(3) $$H=-\nabla V_{m}+H_{b}$$

where $V_{m}$ defined as scalar magnetic potential and $H_{b}$ is the magnetic field intensity of the defined background magnetic field. Thus, the magnetic field intensity equation (*H*) is solved by using the defined scalar magnetic potential ($V_{m}$) and the magnetic field intensity of the defined background magnetic field ($H_{b}$). The background magnetic field can be calculated using the given magnetic flux density *B*

(4) $$H=\frac{B}{\mu_{0}\mu_{r}}$$

where $\mu_{0}$ is the permeability of vacuum and $\mu_{r}$ denotes the relative permeability. By using an “infinite element domain” in COMSOL, a background magnetic field with an infinite area is obtained. The outer boundary of the infinite element domain is set to magnetic isolation by adding “external magnetic flux density” (see also Figure 6):

(5) n·B=0

where *n* represents unit normal. By adding zero magnetic scalar potential, a point at the boundary between the infinite element domain and the silicon oil domain is set as:

(6) $$V_{m}=0$$

Then, Equation (3) is solved and the load is calculated according to the following equations in the “force calculation” in COMSOL.

(7) $$F=\int^{\;}dnTdS$$

(8) $$M=\int^{\;}d\left(r-r_{0}\right)\times \left(nT\right)dS$$

where *T* is the maxwell stress tensor, *n* is the unit outer-pointing normal of the object, *S* is the surface of the object, $r_{0}$ is the pivot point of rotation and *M* is the torque. The fiber rotates in the z-direction in the xy-plane. Therefore, the rotation axis *r*_ax_ = (0, 0, 1) and the pivot point of rotation $r_{0}$ = (0, 0) are set in this work.

In the second stage—the dynamic simulation—the rotation of the fiber in the fluid was simulated. This is relevant for FSI, which represents the interaction of a fluid with a solid. In the present case, the fluid is to be considered as an incompressible fluid. For such incompressible fluids, the continuity equation given in Equation (9) is applied. The Navier–Stokes equation of incompressible laminar flow during injection molding can be found in Equation (10). The Navier–Stokes equation of incompressible creeping flow during press molding processes can be found in Equation (11).

**Figure 7:**
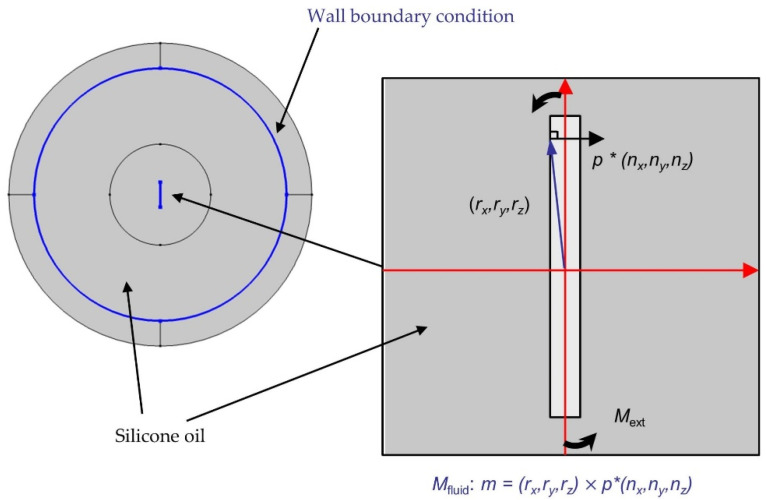
Overview of the specified simulation model, with the individual components considered and the resulting Moment (*M*_ext_) caused by spatial dependent fluid pressure (*p*(*r*_x_, *r*_y_, *r*_z_)).

(9) ρ∇·(u)=0

(10) $$\rho \frac{\partial u}{\partial t}+\rho \left(u\cdot \nabla \right)u=\nabla \cdot \left[-pI_{n}+\eta \left(\nabla u+{\left(\nabla u\right)}^{T}\right)\right]+F$$

(11) $$\rho \frac{\partial u}{\partial t}=\nabla \cdot \left[-pI_{n}+\eta \left(\nabla u+{\left(\nabla u\right)}^{T}\right)\right]+F$$

where ρ is the fluid density, *u* is the flow velocity, *p* is the fluid pressure, *I* is the identity matrix, *η* is the dynamic viscosity and *F* is the external forces. The calculation result of simulation stage 1 is imported into the dynamic simulation as an external force acting on the fiber. As the relation between magnetic torque and rotation angle is sinusoidal in principle, a fitted sinusoidal function is used, since it is continuously differentiable. The force coming from the magnetic field is calculated using the following command:

(12) $$M_{\mathrm{ext}}=an10\left(mod\left(alpha,360\left[deg\right]\right)\right)$$

where *M*_ext_ is the magnetic torque, an is the analytical (in this case sinusoidal) function in COMSOL and *mod* is the modulo operation. For the rotational motion of the fibers in the fluid, the coupling is achieved by manually adding the reaction of the fluid on the fibers (fluid applying torque on the fibers). The resistance of the fluid comes from the fluid pressure *p*. The pressure *p* corresponds to the force per surface. In COMSOL, the normal vector of an edge is defined as (*n*_x_, *n*_y_), the normal vector of a surface is defined as (*n*_x_, *n*_y_, *n*_z_). This force always acts in the normal direction. Therefore, the vector of force density is given by *f* = *p* * (*n*_x_, *n*_y_, *n*_z_). The torque is determined as follows:

(13) M=r×F

where *r* is the vector from the reference point (here the axis of rotation) to the point under consideration and *F* is the force. Analogously, a surface torque density can be defined as follows:

(14) $$m=p*\left(r_{x},\;r_{y},\;r_{z}\right)\times \left(n_{x},\;n_{y},\;n_{z}\right)$$

where *p* is the fluid pressure; *r*_x_, *r*_y_, *r*_z_ denote the spatial coordinates; (*n*_x_, *n*_y_, *n*_z_) is the normal vector of a surface. Because the reference point is the origin (0, 0, 0), the vector (*r*_x_, *r*_y_, *r*_z_) will be equal to (*x*, *y*, *z*) after the calculation (*x*-0, *y*-0, *z*-0). After calculating the cross product, the result is *p* ∗ (*n*_y_ ∗ *x* − *n*_x_ ∗ *y*). To calculate the torque (*M*_fluid_), the force density on the surface of the fiber is integrated (Figure 7).

(15) $$M_{\mathrm{fluid}}=intop1\left(p*\left(n_{y}*x-n_{x}*y\right)\right)*thickness$$

where *M*_fluid_ is the reaction of the fluid on the fiber; *intop*1 is the one-dimensional integral function in COMSOL and the *thickness* is the depth of the model. The operator *intop*1 performs a surface integral of the surface torque density. The integrated expression calculates the torque on the particle in regard to the coordinate origin. The algebraic sign of this expression depends on the direction of the surface normals of the fiber geometry. By integrating this expression over the surface, the total torque is obtained. The variable *thickness* is required to scale the 2D-related torque up to the object’s physical thickness.

## 4. Results and Discussion

### 4.1. Results of the Magnetostatic Simulation

**Figure 8:**
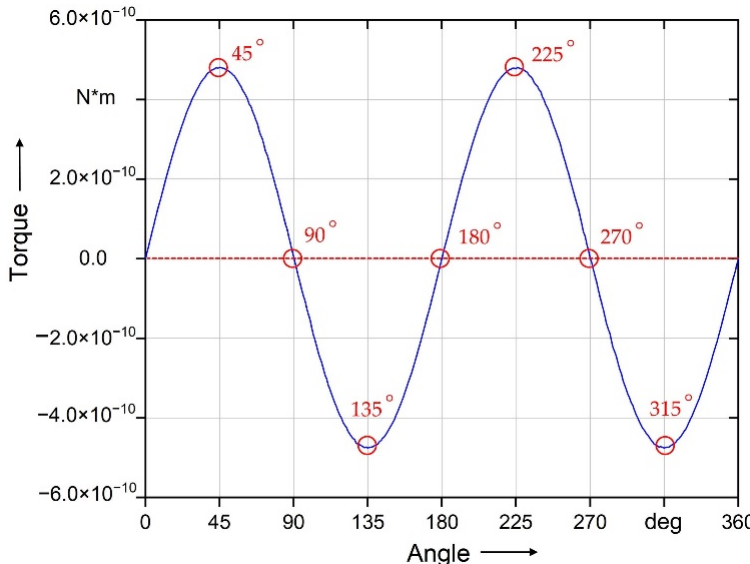
Result of the magnetic torque on the fiber of the short fiber (500 µm) with the coating thickness of 0.2 μm at *B*_0_ = 50 mT.

The fiber was simulated regarding different starting angles in simulation stage 1. The result shows that the relationship between the magnetic torque on the fiber and the angle is represented by a sinusoidal function. The maximum torque on the fiber occurs at the angle of 45°, 135°, 225° and 315°. Furthermore, the minimum torque on the fiber is zero at the angle of 0°, 90°, 180°, 270°, and 360° (Figure 8).

**Figure 9:**
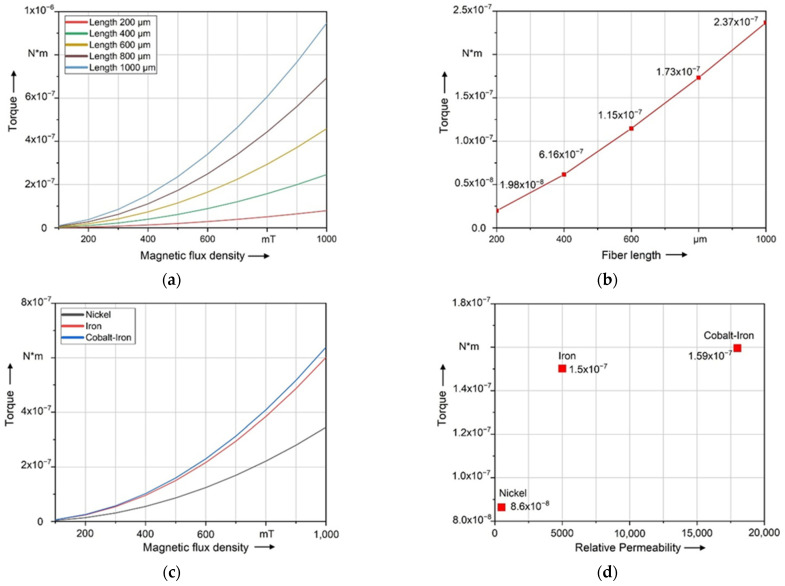
Magnetostatic simulation—resulting magnetic torque (M): (**a**) dependency of fiber lengths at different magnetic flux densities; (**b**) dependency of fiber lengths at a constant magnetic field of *B*_0_ = 500 mT; (**c**) dependency of coating materials/different relative permeabilities (*µ*_r,c_) at different magnetic flux densities; (**d**) resulting magnetic torque depending on coating materials at a constant magnetic field of *B*_0_ = 500 mT.

Furthermore, Figure 9a,b show the results of the magnetostatic simulation for different fiber lengths. As the fiber length increases, the magnetic torque on the fiber also increases. This is because the volume of the coating, which causes the magnetic torque on the fiber by applying the magnetic field, also increases with the fiber length. Figure 9c,d show the result of the magnetostatic simulation for different coating materials. When the relative permeability (*µ*_r,__c_) exceeds 5000 (Iron), the coating material will only affect the increase in the resulting torque to a minor extent. The difference in the magnetic torque between Cobalt–Iron (*µ*_r,__c_ = 18,000) and Iron (*µ*_r,__c_ = 5000) is significantly smaller than the difference between Iron and Nickel (*µ*_r,__c_ = 500). The calculation results point out that there is a quadratic relationship between the magnetic torque and the magnetic flux density *B*_0_.

### 4.2. Results of the Dynamic FSI Simulation

**Figure 10:**
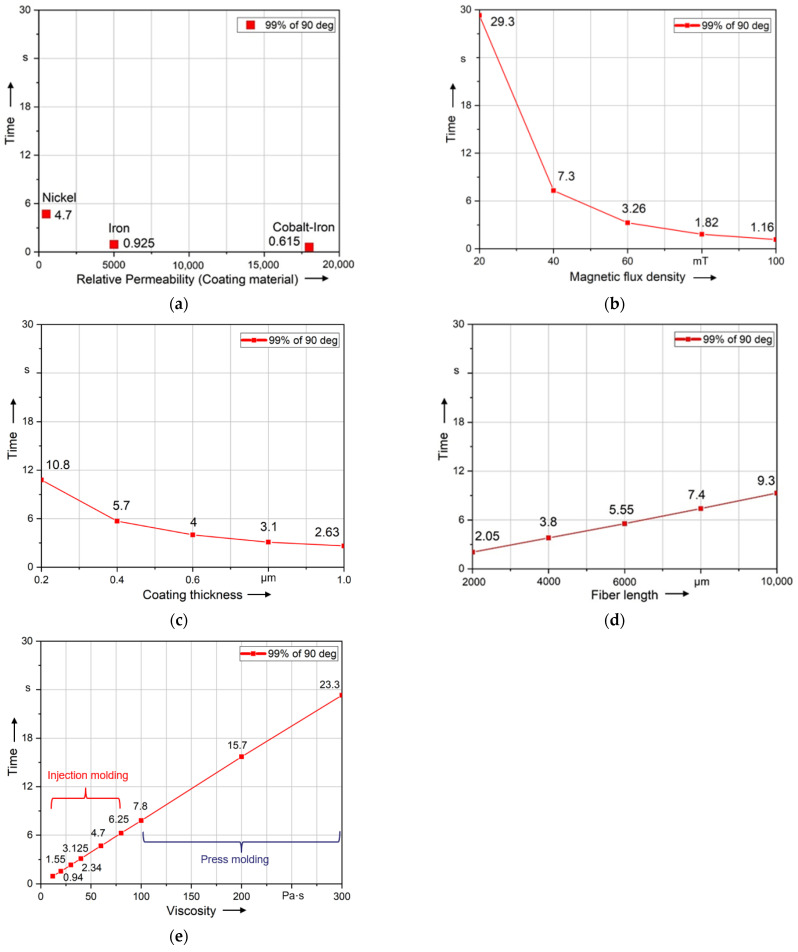
Comparison of the influence of different parameters on the alignment of the long fibers (5000 µm): (**a**) coating material; (**b**) magnetic flux density; (**c**) coating thickness; (**d**) fiber length; (**e**) viscosity.

In this paper, the ability to align fibers is evaluated using the calculated time required to align the fiber by a specified angle. The time of fiber rotation to 89° (99% of 90°) was recorded. Figure 10a–e show this alignment time in regard to the investigated parameters. Compared to all other parameters, the fiber is aligned in the shortest time using Cobalt–Iron coating material. However, the magnetic flux density has the greatest influence on the alignment of the fiber. Compared to all other parameters, the fiber is aligned the slowest at *B*_0_ = 20 mT, but when increasing *B*_0_ to 100 mT, the alignment time is drastically reduced. Thus, the magnetic field variation (*B*_0_) shows the highest impact on the rotation time. In comparison to that, the fiber length and coating thickness show a smaller influence on the time of alignment. In this context, a reduction in the alignment time of 78% results by using a fiber length of 2000 μm compared to the length of 10,000 μm. In regard to the coating thickness, the alignment time can be reduced by 76% comparing a coating thickness of 1 μm to 0.2 μm. The rotation time changes slightly with the change of fiber length and coating thickness. Additionally, the viscosity shows a nearly linear relation to the alignment time, whereby rising viscosity results in rising alignment times. In this context and relation to injection molding processes, a reduction in the alignment time of 85% can be achieved by reducing the viscosity from 80 Pa·s to 12 Pa·s. It can be concluded from Figure 10 that the influence of parameters on the alignment could be sorted in such descending sequence as follows: 1. magnetic flux density; 2. coating material; 3. viscosity during injection molding (12 Pa·s to 80 Pa·s); 4. fiber length; 5. coating thickness; 6. viscosity during press molding (100 Pa·s to 300 Pa·s).

**Figure 11:**
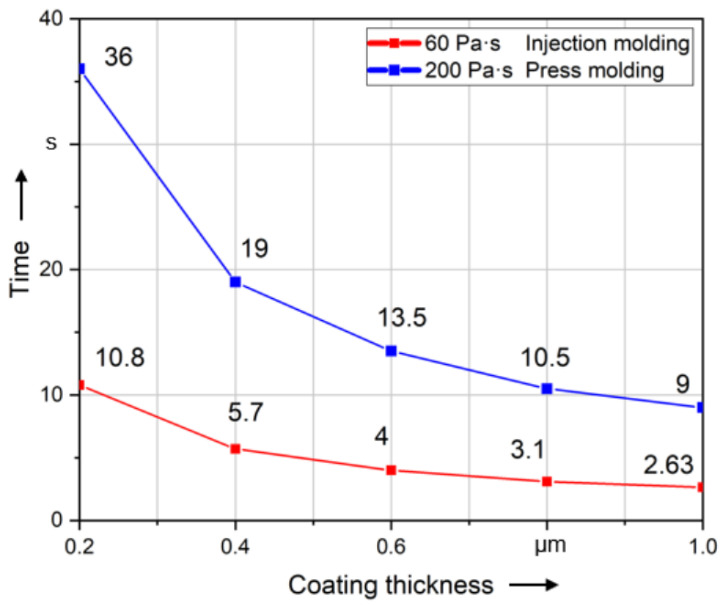
Time required to align long fibers (5000 µm) of different coating thicknesses to 99% of 90°.

Figure 11 shows the influence of the coating thickness on the alignment. The higher the value of coating thickness, the easier it is to align the fiber. There is an approximate exponential functional relationship between the time to alignment and different coating thicknesses. The influence of the coating thickness decreases significantly when it exceeds 0.6 μm.

**Figure 12:**
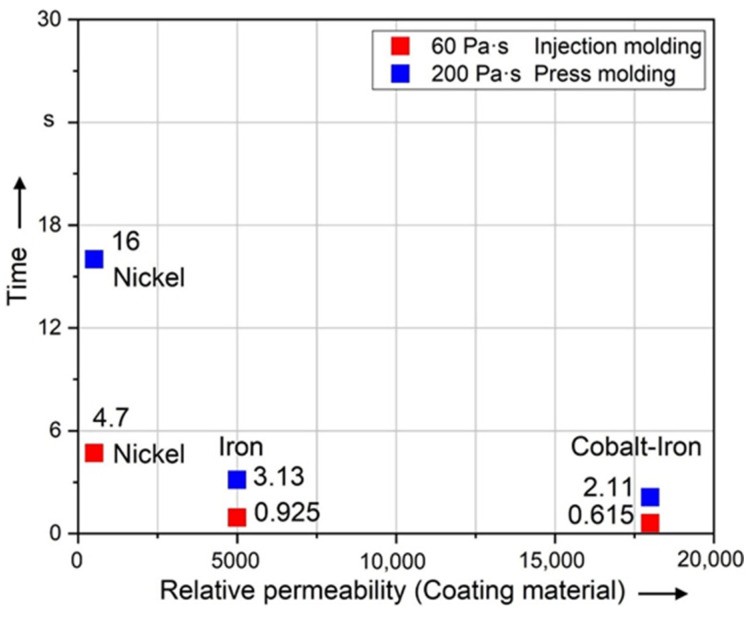
Time required to align long fibers (5000 µm) of different coating materials to 99% of 90°.

Figure 12 shows that the difference between the relative permeability of 5000 (Iron) and 18,000 (Cobalt–Iron) is very small. The magnetostatic simulation concludes that the linear magnetic coating material has little influence on the resulting torque caused by the magnetic field when the relative permeability exceeds 5000 (Iron). The result of the dynamic simulation shown in this diagram corresponds to the result of the magnetostatic simulation. Exceeding a relative permeability of 5000 (Iron), the alignment time will not be significantly affected.

**Figure 13:**
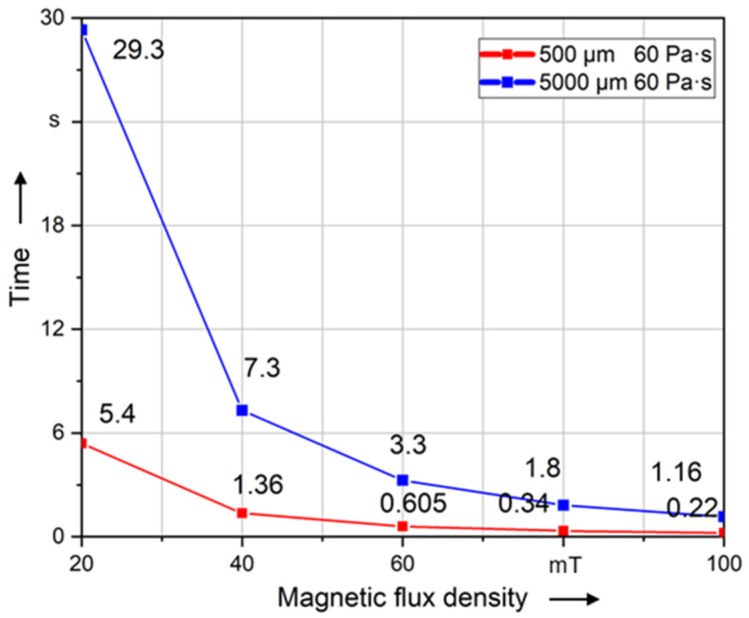
Time required to align short (500 µm) and long fibers (5000 µm) of different magnetic flux densities to 99% of 90° at a constant viscosity.

Figure 13 shows that the time required to align long fiber is more than to align short fiber at the same magnetic flux density. The results of magnetostatic simulation point out that the induced magnetic torque of the fiber increases with the increase in fiber length. However, the interaction of fluid and fiber also increases with the length of the fiber. At the same viscosity, longer fibers are subject to higher resistance and thus require more time. 

**Figure 14:**
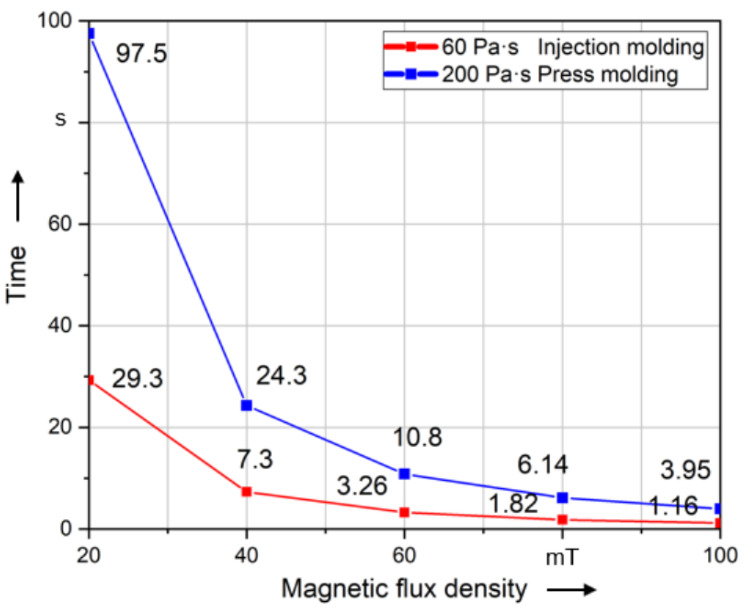
Time required to align long fibers (5000 µm) of different magnetic flux densities to 99% of 90°.

Figure 14 shows that alignment at high *B*_0_ values is much faster than at low magnetic flux values. A slight change in *B*_0_ has a significant effect on the magnetic torque of the magnetic field, which is especially shown when *B*_0_ is less than 60 mT. However, the influence of *B*_0_ decreases significantly when it exceeds 60 mT. In this context, the rotation of a long fiber by 99% of 90° in a time of 1 s using the viscosity of injection molding material requires magnetic flux values higher than 100 mT.

## 5. Conclusions

Fiber-reinforced composites show high potential for lightweight and high-loaded structures. Nevertheless, the fiber orientation is limited by geometric, processing, and material properties. This is especially critical in lightweight structures with high rips or geometric inhomogeneities. For an additional alignment of the reinforcement fibers, magnetic fields seem to be a good possibility. The investigations of the fiber alignment in literature are often performed only for very short fibers or small particles. Thus, the presented paper shows initial investigations for a multiphysics simulation of the alignment process independence of the parameters magnetic flux density, fiber length, coating thickness, the relative permeability of the coating material, and viscosity of the surrounding matrix. Therefore, a coupled two-stage simulation method consisting of static magnetic stimulation and a dynamic FSI simulation was developed, which allows representing the alignment of the fibers and the interaction between fiber and matrix. The magnetostatic simulation (step 1) shows that the resulting magnetic torque and magnetic flux density have a quadratic relationship. For linear magnetic materials, if the relative permeability exceeds 5000 (Iron), the coating material has only little effect on the magnetic torque. Furthermore, the dynamic simulation (step 2) shows that compared to other parameters, the magnetic flux density has the greatest influence on the alignment of the fibers. There is an approximately linear function between the alignment time and the different fiber lengths. The longer the fiber, the slower the rotation. The thicker the coating thickness, the easier it is the alignment of the fibers. The effect of coating thickness is significantly reduced when it exceeds 0.6 μm. The higher the value of the relative magnetic permeability, the easier it is to align the fibers. However, if the relative permeability exceeds 5000 (Iron), the coating material has little effect on the fiber orientation. In the current work, only the rotation of a single fiber is considered. In practice, fibers in a molten polymer not only rotate but also move in either the X or Y direction at the same time. This is a problem in terms of rigid body dynamics. The limitation of considering individual fibers also means that the effect of liquid flow on the rotation of other fibers is not considered. The pressure conditions and flow velocities that would apply in the instrument during injection molding and press molding were initially neglected in the studies listed here. For a possible follow-up study, the task is to simulate the motion of multiple fibers using ordinary differential equations and the FSI interface of COMSOL Multiphysics 5.4. In the present work, the simulations were performed using a 2D model, for possible further research projects, a 3D model could be used instead. Additionally, the effects of fiber interaction, movement and agglomeration will be under further investigation. In order to prevent significant effects of these interfering effects, the approach of pulsed magnetic excitation seems to be target oriented. In the future, this approach will be investigated numerically as well as experimentally.

## Figures and Tables

**Table 1:** Selected silicon oil types and resulting viscosity data for the simulation [23].

Designation	Product Name	Density at 25 °C [g/cm^3^]	Kinematic Viscosity [cst]	Dynamic Viscosity [Pa·s]
S1	ELBESIL SILIKONÖL B 12.500	0.97	12,500	12.125
S2	ELBESIL SILIKONÖL B 20.000	0.97	20,000	19.4
S3	ELBESIL SILIKONÖL B 30.000	0.97	30,000	29.1
S4	ELBESIL SILIKONÖL B 40.000	0.97	40,000	38.8
S5	ELBESIL SILIKONÖL B 60.000	0.97	60,000	58.2
S6	ELBESIL SILIKONÖL B 80.000	0.98	80,000	77.6
S7	SILIKONÖL AK 100.000	0.98	100,000	97
S8	SILIKONÖL AK 200.000	0.98	200,000	194
S9	SILIKONÖL AK 300.000	0.98	300,000	291

**Table 2:** Magnetic permeability of selected materials.

Coating Material	Relative Permeability	Reference
Cobalt–Iron *	18,000	[26]
Iron *	5000	[27]
Steel	100	[28]
Nickel *	100–600	[29,30]
Copper	1	[30]

* Materials used for the parametric studies.

**Table 3:** Parameter data for the simulation.

Parameter	Unit	Range of Values
Viscosity (*η*)	Pa·s	12; 20; 30; 40; 60; 80; 100; 200; 300
Diameter of core carbon fiber (*d*_f_)	μm	7.2
Coating relative permeability (*µ*_r,c_)	-	500, 5000, 18,000
Coating thickness (*t*_c_)	μm	0.2; 0.4; 0.6; 0.8; 1
Fiber length (*l*_f_)	μm	200; 400; 600; 800; 1000; 2000; 4000; 6000; 8000; 10,000
Magnetic field/Magnetic flux density (*B*_0_)	mT	20; 40; 60; 80; 100; 200; 400; 600; 800; 1000

## Data Availability

The data presented in this study are available on request from the corresponding author.

## Nomenclature

*η*	dynamic viscosity
*µ*_r,c_	coating relative permeability
*µ*_0_	permeability of vacuum
*µ*_r_	relative permeability
*ρ*	fluid density
*d*_f_	diameter of core carbon fiber
*t*	rotation time
*t*_c_	coating thickness
*l*_f_	fiber length
*B*_0_	magnetic field/magnetic flux density
*F*	force
*M*	torque
*B*	magnetic flux density
*H*	magnetic field intensity
*V*_m_	scalar magnetic potential
*H*_b_	magnetic field intensity of the defined background magnetic field
*n*	unit outer-pointing normal of the object
*T*	Maxwell stress tensor
*S*	surface of the object
*r*_o_	pivot point of rotation
*r*_ax_	the rotation axis
*u*	flow velocity
*p*	fluid pressure
*I*	identity matrix
*M*_ext_	magnetic torque
*an*	analytical function in COMSOL
*mod*	modulo operation
(*n*_x_, *n*_y_)	normal vector of an edge
(*n*_x_, *n*_y_, *n*_z_)	normal vector of a surface
(*r*_x_, *r*_y_, *r*_z_)	vector from the reference point to the point under consideration
*f*	vector of force density
*m*	surface torque density
*M*_fluid_	reaction of the fluid on the fiber
*intop*	integral function in COMSOL
*thickness*	depth of the model
TFP	tailored fiber placement
NiC	Ni-Coated
PDMS	polydimethylsiloxane
FEM	finite element method
FSI	fluid–structure interaction

## References

[1] Ahmad M.S., Zihilif A.M., Martuscelli E., Ragosta G., Scafora E. (1992). The Electrical Conductivity of Polypropylene and Nickel-Coated Carbon Fiber Composite. Polym. Compos..

[2] Pishvar M., Amirkhosravi M., Altan M. Processing and Alignment of Short Carbon Fibers in an Epoxy Composite by a Magnetic Field. Proceedings of the Americas Conference of the Polymer Processing Society 2018.

[3] Neitzel M., Mitschang P., Breuer U. (2014). Handbuch Verbundwerkstoffe: Werkstoffe, Verarbeitung, Anwendung.

[4] Kim G., Shkel Y.M. (2004). Polymeric Composites Tailored by Electric Field. J. Mater. Res..

[5] Ciambella J., Stanier D.C., Rahatekar S.S. (2017). Magnetic Alignment of Short Carbon Fibres in Curing Composites. Compos. Part B Eng..

[6] Stanier D.C., Ciambella J., Rahatekar S.S. (2016). Fabrication and Characterisation of Short Fibre Reinforced Elastomer Composites for Bending and Twisting Magnetic Actuation. Compos. Part Appl. Sci. Manuf..

[7] Kim H.C., Kim J.W., Zhai L., Kim J. (2019). Strong and Tough Long Cellulose Fibers Made by Aligning Cellulose Nanofibers under Magnetic and Electric Fields. Cellulose.

[8] Scholz M.-S., Drinkwater B.W., Trask R.S. (2014). Ultrasonic Assembly of Anisotropic Short Fibre Reinforced Composites. Ultrasonics.

[9] Timbrell V. (1972). Alignment of Carbon and Other Man-made Fibers by Magnetic Fields. J. Appl. Phys..

[10] Chung C.Y., Chen S.C., Lin K.J. (2018). Effect of Magnetic Field on the Fiber Orientation during the Filling Process in Injection Molding, Part 1: Simulation and Mold Design. Mater. Sci. Forum.

[11] Chen S.C., Chung C.Y., Tseng Y.L. (2018). Effect of Magnetic Field on the Fiber Orientation during the Filling Process in Injection Molding, Part 2: Experiments and Electrical Conductivity Measurements. Mater. Sci. Forum.

[12] Pishvar M., Amirkhosravi M., Altan M.C. Alignment of Nickel Coated Carbon Fibers by Magnetic Field during Cure of Polymer Composites. Proceedings of the American Society for Composites 2018.

[13] Masuda S., Itoh T. (1989). Electrostatic Means for Fabrication of Fiber-Reinforced Metals. IEEE Trans. Ind. Appl..

[14] Itoh T., Masuda S., Gomi F. (1994). Electrostatic Orientation of Ceramic Short Fibers in Liquid. J. Electrost..

[15] Erb R.M., Libanori R., Rothfuchs N., Studart A.R. (2012). Composites Reinforced in Three Dimensions by Using Low Magnetic Fields. Science.

[16] Hatta H., Yamashita S. (1988). Fiber Orientation Control by Means of Magnetic Moment. J. Compos. Mater..

[17] Bordel D., Putaux J.-L., Heux L. (2006). Orientation of Native Cellulose in an Electric Field. Langmuir.

[18] Takeyama S., Nakamura S., Uchida K. (2006). Dynamical Orientation of Carbon Nanotubes by Pulsed Magnetic Fields. J. Phys. Conf. Ser..

[19] Lu G., Li X., Jiang H. (1996). Electrical and Shielding Properties of ABS Resin Filled with Nickel-Coated Carbon Fibers. Compos. Sci. Technol..

[20] Kimura T. (2003). Study on the Effect of Magnetic Fields on Polymeric Materials and Its Application. Polym. J..

[21] DIN 54811-1984-05-Beuth. https://www.beuth.de/de/norm/din-54811/1118726.

[22] Littek S., Schneider M. (2012). Messung zum Materialabbau von Polypropylen. J. Plast. Technol..

[23] QUAX GmbH (2018). Datasheet: Eigenschaften der Standardviskosität.

[24] Zhang L.-Z., Wang X.-J., Quan Y.-Y., Pei L.-X. (2013). Conjugate Heat Conduction in Filled Composite Materials Considering Interactions between the Filler and Base Materials. Int. J. Heat Mass Transf..

[25] Hajjari E., Divandari M., Mirhabibi A. (2004). The Study of Electroless Coating of Nickel on Carbon Fibers. Iran. J. Mater. Sci. Eng..

[26] Vaccumschmelze GmbH (2021). Datasheet: Soft Magnetic Cobalt-Iron Alloys.

[27] Sharma D. (2017). Pure Iron and Low Carbon Steels—Soft Magnetic P/M Materials. J. Chem. Pharm. Res..

[28] Pozo B., Garate J.I., Araujo J.Á., Ferreiro S. (2019). Energy Harvesting Technologies and Equivalent Electronic Structural Models—Review. Electronics.

[29] Chung D.D. (2001). Applied Materials Science: Applications of Engineering Materials in Structural, Electronics, Thermal, and Other Industries.

[30] Paul C.R. (2006). Introduction to Electromagnetic Compatibility.

[31] Schürmann H. (2007). Konstruieren mit Faser-Kunststoff-Verbunden.

